# The identification of high-performing antibodies for Superoxide dismutase [Cu-Zn] 1 (SOD1) for use in Western blot, immunoprecipitation, and immunofluorescence

**DOI:** 10.12688/f1000research.132952.1

**Published:** 2023-04-13

**Authors:** Riham Ayoubi, Walaa Alshafie, Zhipeng You, Kathleen Southern, Peter S. McPherson, Carl Laflamme

**Affiliations:** 1 Department of Neurology and Neurosurgery, Structural Genomics Consortium, The Montreal Neurological Institute, McGill University, Montreal, Québec, H34 2B4, Canada; 2 The Neuro’s Early Drug Discovery Unit (EDDU), Structural Genomics Consortium, McGill University, Montreal, Québec, H3A 2B4, Canada

**Keywords:** Uniprot ID P00441, SOD1, Superoxide dismutase [Cu-Zn], Superoxide dismutase 1, antibody characterization, antibody validation, Western blot, immunoprecipitation, immunofluorescence

## Abstract

Superoxide dismutase [Cu-Zn] 1 (SOD1), is an antioxidant enzyme encoded by the gene *SOD1*, responsible for regulating oxidative stress levels by sequestering free radicals. Identified as the first gene with mutations in Amyotrophic lateral sclerosis (ALS), *SOD1* is a determinant for studying diseases of aging and neurodegeneration. With guidance on well-characterized anti-SOD1 antibodies, the reproducibility of SOD1 research would be enhanced. In this study, we characterized eleven SOD1 commercial antibodies for Western blot, immunoprecipitation, and immunofluorescence using a standardized experimental protocol based on comparing read-outs in knockout cell lines and isogenic parental controls. We identified many high-performing antibodies and encourage readers to use this report as a guide to select the most appropriate antibody for their specific needs.

## Introduction

Superoxide dismutase [Cu/Zn] 1 (SOD1) is an essential enzyme that protects the body against oxidative stress by acting as the first line of defense against reactive oxidative species. ^1^^,^^2^ Largely cytosolic but also found in the mitochondrial intermembrane space, SOD1 is a 153 amino acid protein functioning as a homodimer to bind copper and zinc in order to carry out its role in scavenging free radicals. ^3^^,^^4^

*SOD1* was the first gene in which its mutations were identified in ALS over 30 years ago, predicting it to be a causative factor in motor neuron degeneration. ^5^ A hallmark of SOD1-associated ALS is the misfolding and aggregation of SOD1 into neurotoxic species induced by gene mutations. ^6^ The disease mechanism in which this occurs remains unknown. ^6^ Mechanistic studies would be greatly facilitated with the availability of high-quality antibodies.

Here, we compared the performance of a range of commercially available antibodies for SOD1 and validated several antibodies for Western blot, immunoprecipitation and immunofluorescence, enabling biochemical and cellular assessment of SOD1 properties and function.

## Results and discussion

Our standard protocol involved comparing readouts from wild-type (WT) and knockout (KO) cells. ^7^^–^^9^ To identify a cell line that expressed adequate levels of SOD1 protein to provide sufficient signal to noise, we examined public proteomics databases, namely PaxDB ^10^ and DepMap. ^11^ HeLa was identified as a suitable cell line and thus HeLa was modified with CRISPR/Cas9 to knockout the corresponding *SOD1* gene ( Table 1).

**Table 1.** Summary of the cell lines used.

Institution	Catalog number	RRID (Cellosaurus)	Cell line	Genotype
ATCC	CCL-2	CVCL_0030	HeLa	WT
Montreal Neurological Institute	-	CVCL_A8PZ	HeLa	*SOD1* KO

**Figure 1.**
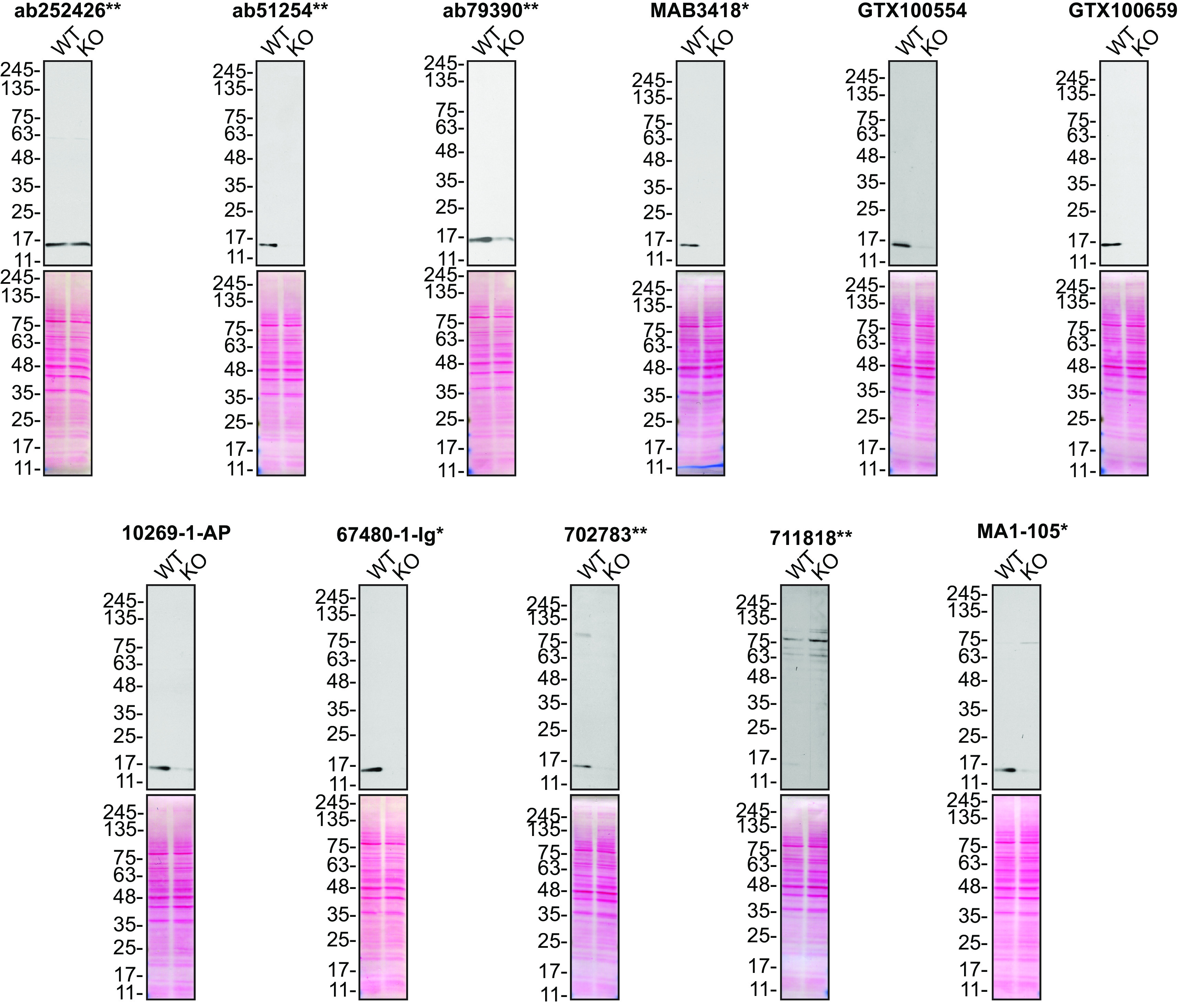
SOD1 antibody screening by Western Blot. Lysates of HeLa (WT and *SOD1* KO) were prepared, and 20 μg of protein were processed for Western blot with the indicated SOD1 antibodies. The Ponceau stained transfers of each blot are presented to show equal loading of WT and KO lysates and protein transfer efficiency from the acrylamide gels to the nitrocellulose membrane. Antibody dilutions were chosen according to the recommendations of the antibody supplier. Exceptions were given for antibodies ab51254 ^**^ and 10269-1-AP, which were titrated to 1/15000 and 1/1000, respectively, as the signals were too weak when following the supplier’s recommendations. Antibody dilution used: ab252426 ^**^ at 1/1000, ab51254 ^**^ at 1/15000, ab79390 ^**^ at 1/10000, MAB3418 ^*^ at 1/1000, GTX100554 at 1/1000, GTX100659 at 1/1000, 10269-1-AP at 1/1000, 67480-1-Ig ^*^ at 1/10000, 702783 ^**^ at 1/200, 711818 ^**^ at 1/200, MA1-105 ^*^ at 1/1000. Predicted band size: 16 kDa. *= monoclonal antibody, **= recombinant antibody.

For Western blot experiments, we resolved proteins from WT and *SOD1* KO cell extracts and probed them side-by-side with all antibodies in parallel ^8^^,^^9^ ( Figure 1). SOD1 is an common essential gene ^12^ and the remaining SOD1 expression in the KO lysate could be detected with various antibodies.

**Figure 2.**
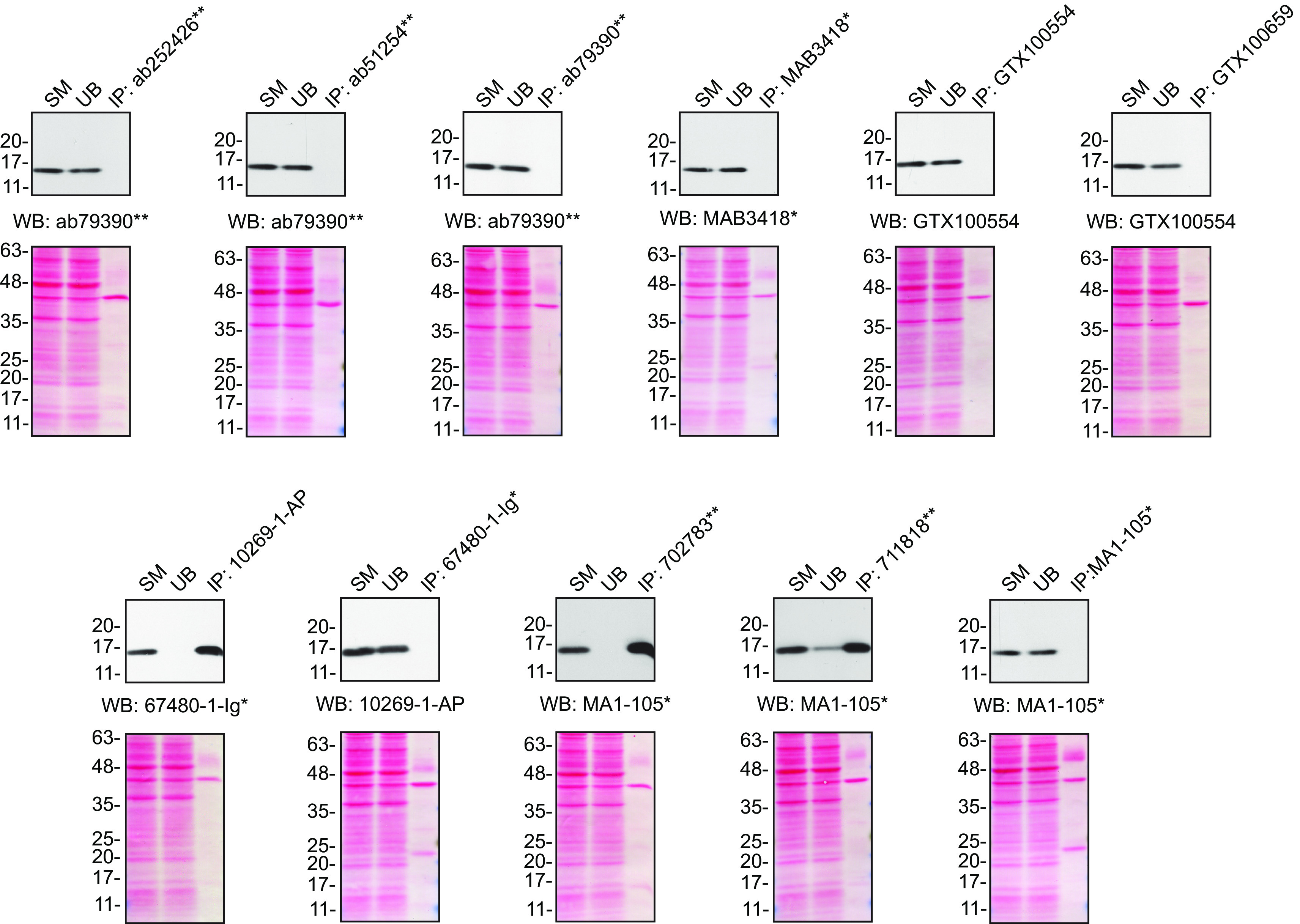
SOD1 antibody screening by immunoprecipitation. HeLa lysates were prepared, and IP was performed using 2.0 μg of the indicated SOD1 antibodies pre-coupled to protein G or protein A Sepharose beads. Samples were washed and processed for Western blot with the indicated SOD1 antibody. For Western blot, MAB3418 ^*^ was used at 1/1000, 67480-1-Ig ^*^ at 1/1000, 10269-1-AP at 1/2000, MA1-105 ^*^ at 1/2000, ab79390 ^**^ at 1/15000 and GTX100554 at 1/2000. The Ponceau stained transfers of each blot are shown for similar reasons as in Figure 1. SM=10% starting material; UB=10% unbound fraction; IP=immunoprecipitate; *= monoclonal antibody; **= recombinant antibody.

For immunoprecipitation experiments, we used the antibodies to immunopurify SOD1 from HeLa cell extracts. The performance of each antibody was evaluated by detecting the SOD1 protein in extracts, in the immunodepleted extracts and in the immunoprecipitates ^8^^,^^9^ ( Figure 2).

**Figure 3.**
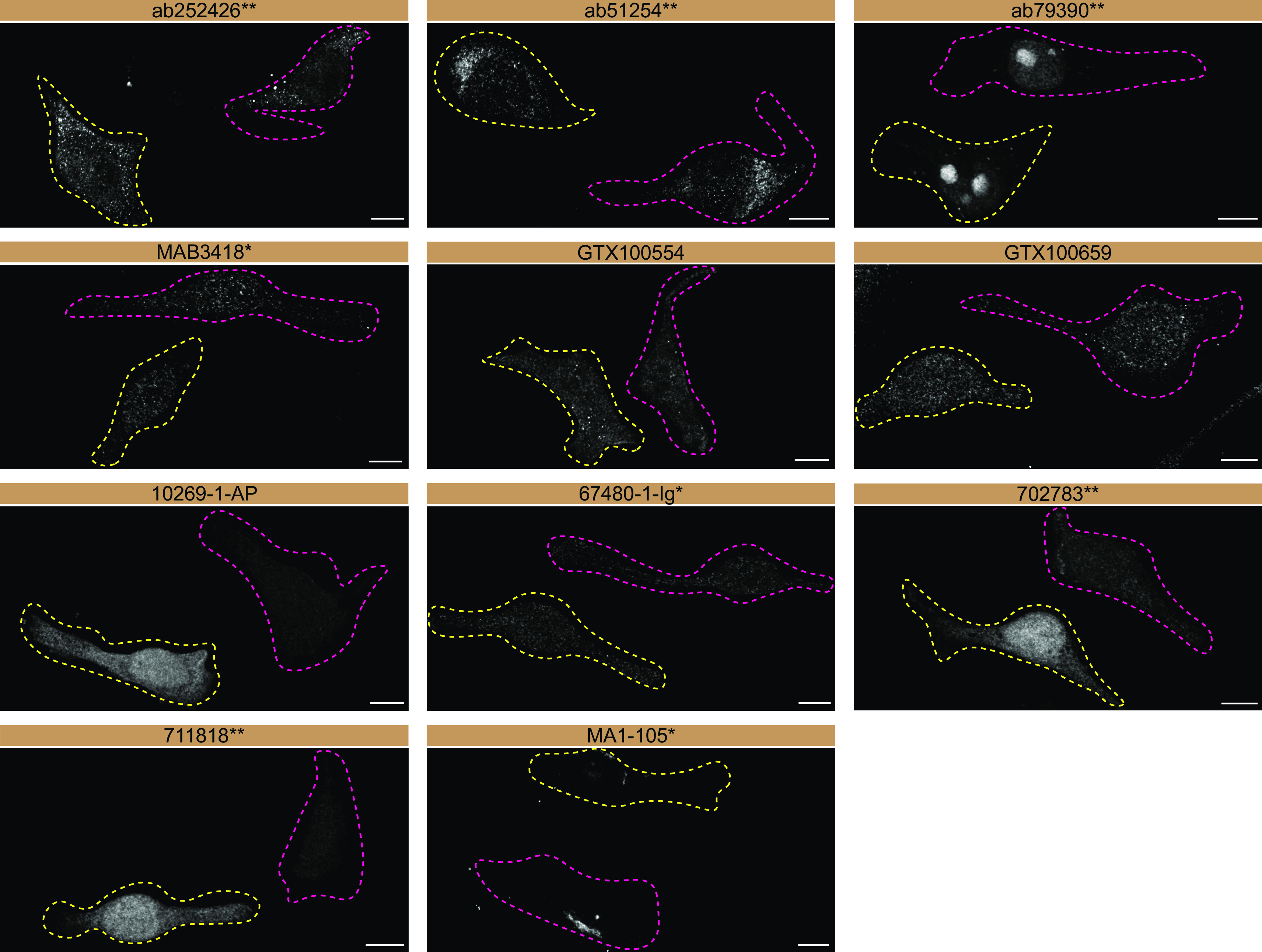
SOD1 antibody screening by immunofluorescence. HeLa WT and *SOD1* KO cells were labelled with a green or a far-red fluorescent dye, respectively. WT and KO cells were mixed and plated to a 1:1 ratio on coverslips. Cells were stained with the indicated SOD1 antibodies and with the corresponding Alexa-fluor 555 coupled secondary antibody. Acquisition of the green (identification of WT cells), red (antibody staining) and far-red (identification of KO cells) channels was performed. Representative images of the red (grayscale) channel are shown. WT and KO cells are outlined with yellow and magenta dashed line, respectively. Antibody dilutions were chosen according to the recommendations of the antibody supplier. An exception was given for antibody 10269-1-AP, which was titrated to 1/500, as the signal was too weak when following the supplier’s recommendations. When the concentration was not indicated by the supplier, which was the case for ab252426 ^**^, GTX100554, GTX100659, 702783 ^**^, 711818 ^**^ and MA1-105*, we tested antibodies at 1/200, 1/500 or 1/1000. At these concentrations, the signal from each antibody was in the range of detection of the microscope used. Antibody dilution used: ab252426 ^**^ at 1/500, ab51254 ^**^ at 1/200, ab79390 ^**^ at 1/200, MAB3418 ^*^ at 1/500, GTX100554 at 1/200, GTX100659 at 1/500, 10269-1-AP at 1/500, 67480-1-Ig ^*^ at 1/500, 702783 ^**^ at 1/500, 711818 ^**^ at 1/500, MA1-105 ^*^ at 1/1000. Bars = 10 μm. *= monoclonal antibody; **= recombinant antibody.

For immunofluorescence, as described previously, antibodies were screened using a mosaic strategy. ^13^ In brief, we plated WT and KO cells together in the same well and imaged both cell types in the same field of view to reduce staining, imaging and image analysis bias ( Figure 3).

In conclusion, we have screened SOD1 commercial antibodies by Western blot, immunoprecipitation and immunofluorescence and identified several high-quality antibodies under our standardized experimental conditions. The underlying data can be found on Zenodo. ^14^^,^^15^

## Methods

### Antibodies

All SOD1 antibodies are listed in Table 2, together with their corresponding Research Resource Identifiers, or RRID, to ensure the antibodies are cited properly. ^16^ Peroxidase-conjugated goat anti-rabbit and anti-mouse antibodies are from Thermo Fisher Scientific (cat. number 65-6120 and 62-6520). Alexa-555-conjugated goat anti-mouse and anti-rabbit secondary antibodies are from Thermo Fisher Scientific (cat. number A21424 and A21429).

**Table 2.** Summary of the SOD1 antibodies tested.

Company	Catalog number	Lot number	RRID (Antibody Registry)	Clonality	Clone ID	Host	Concentration (μg/μL)	Vendors recommended applications
Abcam	ab252426 **	GR3334282-1	AB_2885125	recombinant-mono	EPR23549-163	rabbit	0.48	Wb, IP, IF
Abcam	ab51254 **	GR3231443-1	AB_882757	recombinant-mono	EP1727Y	rabbit	0.15	Wb, IF
Abcam	ab79390 **	GR221266-10	AB_1603741	recombinant-mono	EPR1726	rabbit	0.18	Wb
Bio-Techne	MAB3418 *	XJQ0216121	AB_2193899	monoclonal	348808	mouse	0.50	Wb, IF
GeneTex	GTX100554	43222	AB_10618670	polyclonal	-	rabbit	0.15	Wb, IF
GeneTex	GTX100659	41822	AB_1951972	polyclonal	-	rabbit	0.47	Wb, IF
Proteintech	10269-1-AP	00069112	AB_2193750	polyclonal	-	rabbit	0.43	Wb, IP, IF
Proteintech	67480-1-Ig *	10014544	AB_2882707	monoclonal	2F10G1	mouse	0.50	Wb
Thermo Fisher Scientific	702783 **	2107589	AB_2716893	recombinant-mono	11H3L1	rabbit	0.50	Wb, IF
Thermo Fisher Scientific	711818 **	SH256097	AB_2688303	recombinant-poly	-	rabbit	0.50	Wb, IF
Thermo Fisher Scientific	MA1-105 *	VL315171	AB_2536811	monoclonal	8B10	mouse	1.00	Wb, IF

Wb=Western blot; IF= immunofluorescence; IP=immunoprecipitation;

* = monoclonal antibody;

** = recombinant antibody.

### CRISPR/Cas9 genome editing

HeLa *SOD1* KO clone was generated with low passage cells using an open-access protocol available on Zenodo. Two guide RNAs were used to introduce a STOP codon in the *SOD1* gene (sequence guide 1: CCGTTGCAGTCCTCGGAACC, sequence guide 2: GCGCGGGGGGACGAGCGGGT).

### Cell culture

Both HeLa WT and *SOD1* KO cell lines used are listed in Table 1, together with their corresponding RRID, to ensure the cell lines are cited properly. ^17^ Cells were cultured in DMEM high-glucose (GE Healthcare cat. number SH30081.01) containing 10% fetal bovine serum (Wisent, cat. number 080450), 2 mM L-glutamate (Wisent cat. number 609065), 100 IU penicillin and 100 μg/mL streptomycin (Wisent cat. number 450201).

### Antibody screening by Western Blot

Western blots were performed as described in our standard operating procedure. ^18^ HeLa WT and *SOD1* KO were collected in RIPA buffer (50 mM Tris-HCl pH 8.0, 150mM NaCl, 1.0 mM EDTA, 1% Triton X-100, 0.5% sodium deoxycholate, 0.1% SDS) supplemented with 1x protease inhibitor cocktail mix (MilliporeSigma, cat. number 78429). Lysates were sonicated briefly and incubated for 30 min on ice. Lysates were spun at ~110,000 x g for 15 min at 4°C and equal protein aliquots of the supernatants were analyzed by SDS-PAGE and Western blot. BLUelf prestained protein ladder from GeneDireX (cat. number PM008-0500) was used.

Western blots were performed with large 8-16% polyacrylamide gels and transferred on nitrocellulose membranes. Proteins on the blots were visualized with Ponceau S staining (Thermo Fisher Scientific, cat. number BP103-10) which is scanned to show together with individual Western blot. Blots were blocked with 5% milk for 1 hr, and antibodies were incubated overnight at 4°C with 5% bovine serum albumin (BSA) (Wisent, cat. number 800-095) in TBS with 0,1% Tween 20 (TBST) (Cell Signaling Technology, cat. number 9997). Following three washes with TBST, the peroxidase conjugated secondary antibody was incubated at a dilution of ~0.2 μg/mL in TBST with 5% milk for 1 hr at room temperature followed by three washes with TBST. Membranes were incubated with Pierce ECL (Thermo Fisher Scientific, cat. number 32106) prior to detection with the HyBlot CL autoradiography films (Denville, cat. number 1159T41).

### Antibody screening by immunoprecipitation

Immunoprecipitation was performed as described in our standard operating procedure. ^19^ Antibody-bead conjugates were prepared by 2.0 μg of antibody to 500 μL of phosphate-buffered saline (PBS) (Wisent, cat. number 311-010-CL) with 0,01% triton X-100 (Thermo Fisher Scientific, cat. number BP151-500) in a 1.5 mL microcentrifuge tube, together with 30 μL of protein A- (for rabbit antibodies) or protein G- (for mouse antibodies) Sepharose beads. Tubes were rocked overnight at 4°C followed by two washes to remove unbound antibodies.

HeLa WT were collected in HEPES lysis buffer (20 mM HEPES, 100 mM sodium chloride, 1 mM EDTA, 1% Triton X-100, pH 7.4) supplemented with protease inhibitor. Lysates were rocked 30 min at 4°C and spun at 110,000 x g for 15 min at 4°C. One mL aliquots at 0.5 mg/mL of lysate were incubated with an antibody-bead conjugate for ~2 hours at 4°C. The unbound fractions were collected, and beads were subsequently washed three times with 1.0 mL of HEPES lysis buffer and processed for SDS-PAGE and Western blot on 8-16% polyacrylamide gels, as described above. Prot-A:HRP (MilliporeSigma, cat. number P8651) and VeriBlot for IP Detection Reagent HRP (Abcam, cat. number ab131366) were used as secondary detection systems for an experiment where a rabbit antibody was used for both immunoprecipitation and its corresponding Western blot. Similarly, anti- mouse IgG for IP HRP (Abcam, cat. number ab131368) was used for an experiment where a mouse antibody was used for immunoprecipitation and it’s corresponding Western blot.

### Antibody screening by immunofluorescence

Immunofluorescence was performed as described in our standard operating procedure. ^8^^,^^9^^,^^13^ HeLa WT and *SOD1* KO were labelled with a green and a far-red fluorescence dye, respectively. The fluorescent dyes used are from Thermo Fisher Scientific (cat. number C2925 and C34565). WT and KO cells were plated on glass coverslips as a mosaic and incubated for 24 hrs in a cell culture incubator at 37 ^o^C, 5% CO _2_. Cells were fixed in 4% paraformaldehyde (PFA) (Beantown chemical, cat. number 140770-10ml) in PBS for 15 min at room temperature and then washed 3 times with PBS. Cells were permeabilized in PBS with 0,1% Triton X-100 for 10 min at room temperature and blocked with PBS with 5% BSA, 5% goat serum (Gibco, cat. number 16210-064) and 0.01% Triton X-100 for 30 min at room temperature. Cells were incubated with IF buffer (PBS, 5% BSA, 0,01% Triton X-100) containing the primary SOD1 antibodies overnight at 4 °C. Cells were then washed 3 × 10 min with IF buffer and incubated with corresponding Alexa Fluor 555-conjugated secondary antibodies in IF buffer at a dilution of 1.0 μg/mL for 1 hr at room temperature. Cells were washed 3 × 10 min with IF buffer and once with PBS. Coverslips were mounted on a microscopic slide using fluorescence mounting media (DAKO).

Imaging was performed using a Zeiss LSM 880 laser scanning confocal microscope equipped with a Plan-Apo 40x oil objective (NA = 1.40). Analysis was done using the Zen navigation software (Zeiss). All cell images represent a single focal plane. Figures were assembled with Adobe Photoshop (version 24.1.2) to adjust contrast then assembled with Adobe Illustrator (version 27.3.1).

## Data Availability

Zenodo: Antibody Characterization Report for Superoxide Dismutase [Cu-Zn] (SOD1), https://doi.org/10.5281/zenodo.5061103. ^14^ Zenodo: Dataset for the Superoxide Dismutase 1 Cu-Zn (SOD1) antibody screening study, https://doi.org/10.5281/zenodo.7709943. ^15^ Data are available under the terms of the Creative Commons Attribution 4.0 International license (CC-BY 4.0).
