# Supplementary material for: Identification of high-performing antibodies for Superoxide dismutase [Cu-Zn] 1 (SOD1) for use in Western blot, immunoprecipitation, and immunofluorescence
Source: F1000Res. 2023 Aug 31;12:391. Originally published 2023 Apr 13. [Version 2] doi: 10.12688/f1000research.132952.2 (PMC10582621; doi:10.12688/f1000research.132952.2)
Supplement: Peer review history

# Reviewer report: Reviewer response for version 2

**DOI:** 10.5256/f1000research.155278.r214146

**Published:** 2023-10-17

**Authors:** Chi Kwan Tsang

**Affiliations:** 1 Clinical Neuroscience Institute, The First Affiliated Hospital of Jinan University, Guangzhou, China

In this Data Note, Ayoubi et al. tested eleven commercially available SOD1 antibodies in WT and CRISPR/Cas9-mediated knockout HeLa cells. They presented a comprehensive comparisons among these antibodies in Western blot, immunoprecipitation, and immunofluorescence staining using a well-defined experimental protocols. They identified some high-performing antibodies which seem to be appropriate for these experiments while a few other of them failed to produce satisfactory results. This report should be useful for readers who plan to perform the above experiments for selection of suitable antibodies.

        In general, the report was well-written and a few comments and recommendation are listed below:

1. Please carefully check the whole text for typos, e.g. “… an common …”; “An earlier version of this of this article…”.
2. In the reference section, it may be better to add the ULR links for the papers. For example,  they may add “ https://zenodo.org/record/5717516/files/2021-11-21-SOP-IP.pdf?download=1” to ref#19. Same for references #13, 14, 15, 18.
3. In the first paragraph of the Introduction, the authors mentioned that Sod1 is largely cytosolic but also found in the mitochondrial intermembrane space…”. Sod1 is also present in the nucleus. Please add the citation below and clarify this point.
  Tsang CK, Liu, Y., Thomas, J., Zhang, Y., Zheng, X.F. (2014) Superoxide dismutase 1 acts as a nuclear transcription factor to regulate oxidative stress resistance. ***Nature Communications*** *5, 3446. *

Are sufficient details of methods and materials provided to allow replication by others?

Yes

Is the rationale for creating the dataset(s) clearly described?

Yes

Are the datasets clearly presented in a useable and accessible format?

Yes

Are the protocols appropriate and is the work technically sound?

Yes

Reviewer Expertise:

Molecular and Cellular Biology, Molecular Neuroscience, CNS diseases, Stroke.

I confirm that I have read this submission and believe that I have an appropriate level of expertise to confirm that it is of an acceptable scientific standard.

## References

[1] : Superoxide dismutase 1 acts as a nuclear transcription factor to regulate oxidative stress resistance. *Nat Commun* .2014;5: 10.1038/ncomms4446 3446 10.1038/ncomms4446 24647101PMC4678626

# Reviewer report: Reviewer response for version 2

**DOI:** 10.5256/f1000research.155278.r202703

**Published:** 2023-09-07

**Authors:** Nishant N Vaikath

**Affiliations:** 1 Neurological Disorder Research Center, Qatar Biomedical Research Institute (QBRI), Hamad Bin Khalifa University, Doha, Doha, Qatar

Thank you for the revised manuscript. I have no further comments to make.

Are sufficient details of methods and materials provided to allow replication by others?

Yes

Is the rationale for creating the dataset(s) clearly described?

Yes

Are the datasets clearly presented in a useable and accessible format?

Yes

Are the protocols appropriate and is the work technically sound?

Yes

Reviewer Expertise:

Neuroscience, Antibody development, Antibody engineering, Nanobody

I confirm that I have read this submission and believe that I have an appropriate level of expertise to confirm that it is of an acceptable scientific standard.

# Reviewer report: Reviewer response for version 1

**DOI:** 10.5256/f1000research.145911.r186895

**Published:** 2023-08-18

**Authors:** Nishant N Vaikath

**Affiliations:** 1 Neurological Disorder Research Center, Qatar Biomedical Research Institute (QBRI), Hamad Bin Khalifa University, Doha, Doha, Qatar

In this study, Ayoubi et al, have characterized commercially available SOD1 antibodies for use in biochemical techniques, including Western Blotting, Immunoprecipitation, and Immunofluorescence, utilizing lysates from knockout cell lines and isogenic parental controls. The aim was to identify high-performing antibodies suitable for specific applications. While the authors have screened and compared the antibodies using different applications, there are some areas that require improvement, as outlined below:

1. Quantification of HeLa lysates: The authors need to provide information on how the HeLa lysates were quantified. Adding details about the quantification method will enhance the reroducibility and accuracy of the results.
2. Elaboration of the results section: The results section needs to be more detailed and informative. Currently, it lacks clarity regarding the outcomes for each technique and the identification of the best-suited antibodies for each application.
3. Table of tested antibodies: To facilitate better understanding for readers, the authors should consider providing a table that lists the antibodies tested along with the techniques they were evaluated in. This table could highlight which antibodies performed best for each technique, making it easier for researchers to identify suitable antibodies for their experiments.

By addressing these minor comments and enhancing the results section, the study's findings will become more accessible and beneficial to the scientific community.

Are sufficient details of methods and materials provided to allow replication by others?

Yes

Is the rationale for creating the dataset(s) clearly described?

Yes

Are the datasets clearly presented in a useable and accessible format?

Yes

Are the protocols appropriate and is the work technically sound?

Yes

Reviewer Expertise:

Neuroscience, Antibody development, Antibody engineering, Nanobody

I confirm that I have read this submission and believe that I have an appropriate level of expertise to confirm that it is of an acceptable scientific standard, however I have significant reservations, as outlined above.

# Response

**Published:** 2023-08-28

**Authors:** Kathleen Southern

**Affiliations:** McGill University, Canada

Thank you to Nishant N Vaikath for reviewing this article and providing a peer-review report.

 To respond to your first suggestion, we have added a short description of the quantification method used. Please review the Western Blot methods section of the second version of the article we have uploaded.

 As for your second and third points, YCharOs does not engage in result analysis nor do we offer explicit antibody recommendations. The primary goal of our initiative is to deliver high-quality antibody validation data to the scientific community. We have found that, for the most part, scientists viewing our articles have the expertise to interpret the characterization data independently, enabling them to make informed choices regarding the most suitable antibodies for their specific experimental needs. Recognizing the potential ambiguity of this point within the article, we have taken proactive measures to rectify the situation. As such,  a new paragraph has been added to the Results and Discussion section, explaining our reasonings as to why we do not recommend or score the antibodies tested.

 Thank you again for your suggestions, we always appreciate feedback on how to improve the reproducibility of our data. After reviewing the second version of the article, newly submitted, we hope that you chose to reconsider your approval status.
